# The beetle elytron plate: a lightweight, high-strength and buffering functional-structural bionic material

**DOI:** 10.1038/s41598-017-03767-w

**Published:** 2017-06-30

**Authors:** Xiaoming Zhang, Juan Xie, Jinxiang Chen, Yoji Okabe, Longcheng Pan, Mengye Xu

**Affiliations:** 1 Key Laboratory of Concrete and Prestressed Concrete Structures of the Ministry of Education, Southeast University, Nanjing, 210096 China; 2 State Key Laboratory of Subtropical Building Science, South China University of Technology, Guangzhou, 510640 China; 3 Department of Mechanical and Biofunctional Systems, Institute of Industrial Science, The University of Tokyo, 4-6-1 Komaba, Meguro-ku, Tokyo 153-8505 Japan

## Abstract

To investigate the characteristics of compression, buffering and energy dissipation in beetle elytron plates (BEPs), compression experiments were performed on BEPs and honeycomb plates (HPs) with the same wall thickness in different core structures and using different molding methods. The results are as follows: 1) The compressive strength and energy dissipation capacity in the BEP are 2.44 and 5.0 times those in the HP, respectively, when the plates are prepared using the full integrated method (FIM). 2) The buckling stress is directly proportional to the square of the wall thickness (t). Thus, for core structures with equal wall thicknesses, although the core volume of the BEP is 42 percent greater than that of the HP, the mechanical properties of the BEP are several times higher than those of the HP. 3) It is also proven that even when the single integrated method (SIM) is used to prepare BEPs, the properties discussed above remain superior to those of HPs by a factor of several; this finding lays the foundation for accelerating the commercialization of BEPs based on modern manufacturing processes.

## Introduction

The collisions and impacts that occur in various disasters pose a tremendous threat to the safety of human lives and property. Therefore, different structures^1–3^ that offer high energy dissipation capabilities are continuously being sought for their properties of crashworthiness and impact resistance. For this purpose, the honeycomb plate (HP), as a lightweight, high-strength structure^4–6^, exhibits good buffering performance and has found many applications in the fields of aerospace^7–9^, transportation^10^, and architecture^11–13^. The biological prototype for a HP is a honeycomb. Commercially available honeycomb sandwich plates are currently manufactured by adhesively or mechanically joining their skin and core components, which are produced separately using different processes^14^. However, as an alternative inspiration from nature, various species of beetles have survived since the age of the dinosaurs. Thus, after their long process of evolution, beetle elytra possess a remarkable biological structure that exhibits several unique biological functions. For example, the structural color is a typical instance of the combination of a subtle fine structure and camouflage^15–17^, which derives from surfaces or exocuticle of beetle elytra. In fact, the inner three-dimensional structure of elytra and its corresponding biological functions, which not only facilitate flight but also must protect the main body of the insect, can be understood to be an advanced evolutionary biological structure that is both light in weight and high in strength and exhibits excellent buffering performance^18–20^. Therefore, our group has performed bionic studies of beetle elytra from the end of the last century and has found that their structure is composed of a fully integrated honeycomb-trabecular plate (FIHP) with edge-sealing features^21^. Based on these observations, our group has developed a reasonably complete preparation technique for FIHPs^22^ and has revealed that this kind of new sandwich plate possesses excellent mechanical properties^23^.

In our previous paper^24^, from the mechanical perspective, we discovered the reason for material composite of trabeculae and the shared mechanism of trabecular-honeycomb structure (THS) in beetle elytra. Based on these findings, we renamed our FIHPs as beetle elytron plates (BEPs) to express our respect to the biologic structure in beetle. What’s more, it has been proven that a BEP, with hollow trabeculae, possesses better compressive properties compared with a HP for the same volume of the core structure^25^. However, it is general that, in the entire plate, the proportion of the material that makes up the core structure is very small. Thus, for the same wall thickness of the core structures, the difference between the material costs for these two types of plates is also very small. However, the mechanical properties of BEPs are markedly improved over those of HPs, enabling an enormous contribution to in engineering applications. Therefore, the compressive properties of these two different plates were investigated in this study for core structures of the same wall thickness (t). Simultaneously, the influence of single integrated method (SIM) on the compressive properties of BEPs was investigated to accelerate the process of commercialization by utilizing the manufacturing methods developed for HPs.

## Experimental and Modeling Methods

### Background: the origin of the BEP

**Figure 1:**
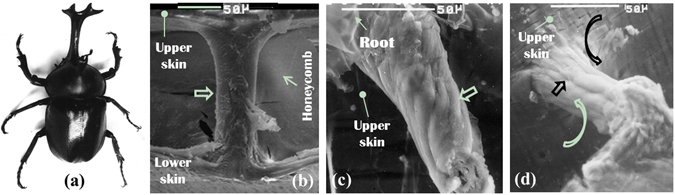
The beetle and microstructure of trabeculae. (**a**) An adult A. dichotoma beetle^21^. (**b**) The shape of a trabecula^18^. (**c**,**d**) The orientations of the chitin fibers on the outside of a trabecula^18^. Trabeculae as indicated by broad arrows.

**Figure 2:**
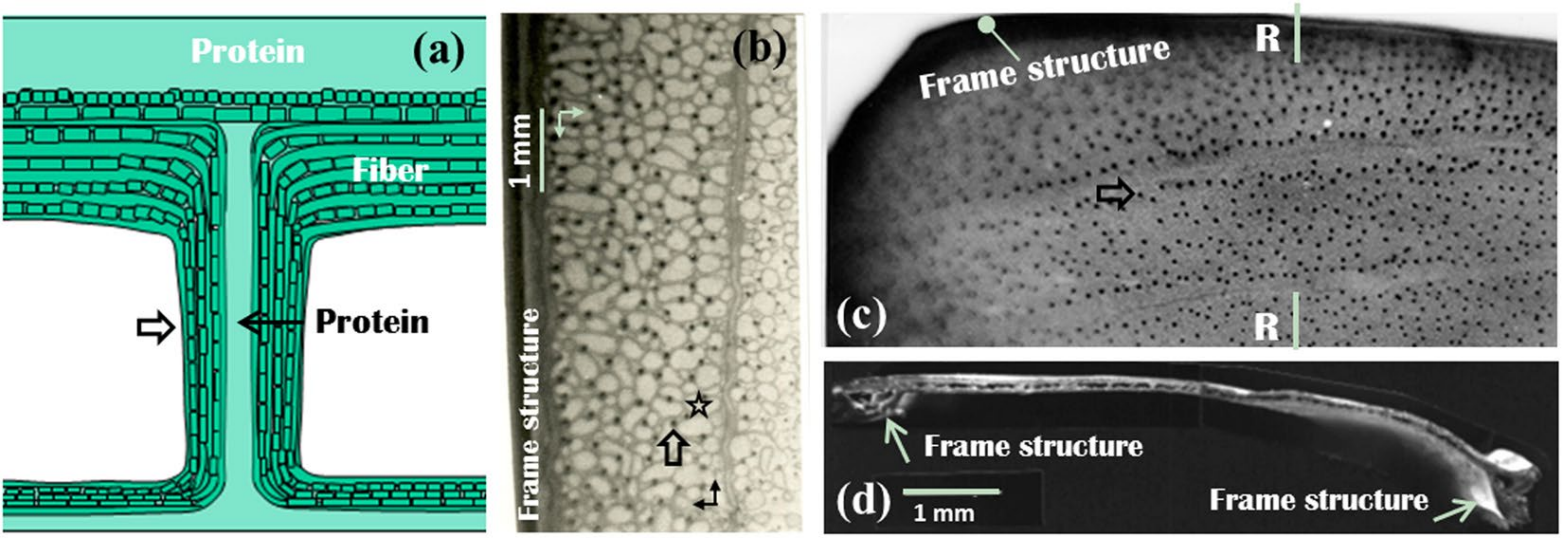
A simple model and the microstructure of beetle elytra. (**a**) A simple model of a trabecula^18^. (**b**) The location distribution of trabeculae in the fresh elytron of A. dichotoma^26^. (**c**) The structure of an elytron after treatment with 10% KOH^18^. (**d**) A cross-sectional view^21^ of section R–R in (**c**). Trabeculae as indicated by broad arrows.

To facilitate the reader’s understanding, the biological prototypes for the BEPs are shown in Figs 1 and 2. The elytron of an adult A. dichotoma beetle (Fig. 1a) consists of upper and lower skins sandwiching a trabecular-honeycomb structure (THS; Fig. 1b). It has been proven that the outermost fibers of the trabeculae are arranged in either a spiral or a linear manner along the trabecular axis to connect with the fibers of the upper and lower skins, with which they exhibit a natural organic integrity (Fig. 1c,d). Furthermore, chitin fibers with a high load-bearing capacity are arranged on the outside of the trabeculae, whereas protein materials with a low load-bearing capacity are found in their centers (Fig. 2a). These connections continuously and organically spread among hundreds to more than thousands of trabeculae (Fig. 2b,c), forming a natural frame structure (Fig. 2d). Based on these observations, JX Chen were the first to propose a biomimetic integrated trabecular-honeycomb sandwich plate structure^18^.

### Model design and experimental methods

**Figure 3:**
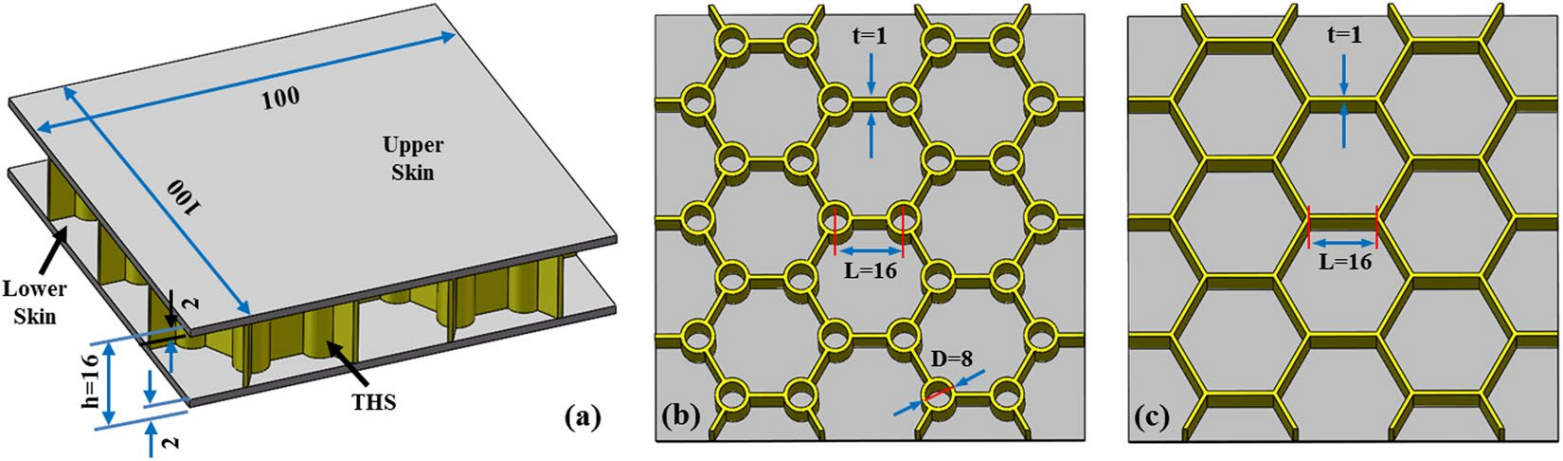
Dimensions and structures of the sandwich plates. (**a**) Overall dimensions. (**b**) core structure of a BEP. (**c**) core structure of a HP. In the figure, L denotes the length of the honeycomb walls or the distance between the central points in adjacent trabeculae, D denotes the external diameter of the trabeculae, the thickness (t) of the honeycomb walls is equal to that of the trabeculae, and the unit is mm.

The earlier paper discussed the differences in the properties of compression, buffering and dissipation between BEPs and HPs when the two plates have the same core volume (V) but different wall thicknesses (t) (hereafter referred to as the equal-volume, or EV, case)^25^. By contrast, for the purposes stated in the introduction, the BEPs and HPs investigated in this study were designed to have the same wall thickness (t) but different core volumes (hereafter referred to as the equal-thickness, or ET, case). The overall dimensions of the two test models are shown in Fig. 3(a); note that the core structures have the same height (h). The detailed dimensions are shown in Fig. 3(b,c). Both types of test models were produced via both the FIM and the SIM (in the latter case, with the upper skin and core structure integrated together and the lower skin join to the core structure with glue (Ergo 5910–1, Switzerland)), and the influence of the different molding methods on the compressive properties of the BEPs and HPs were investigated.

**Figure 4:**
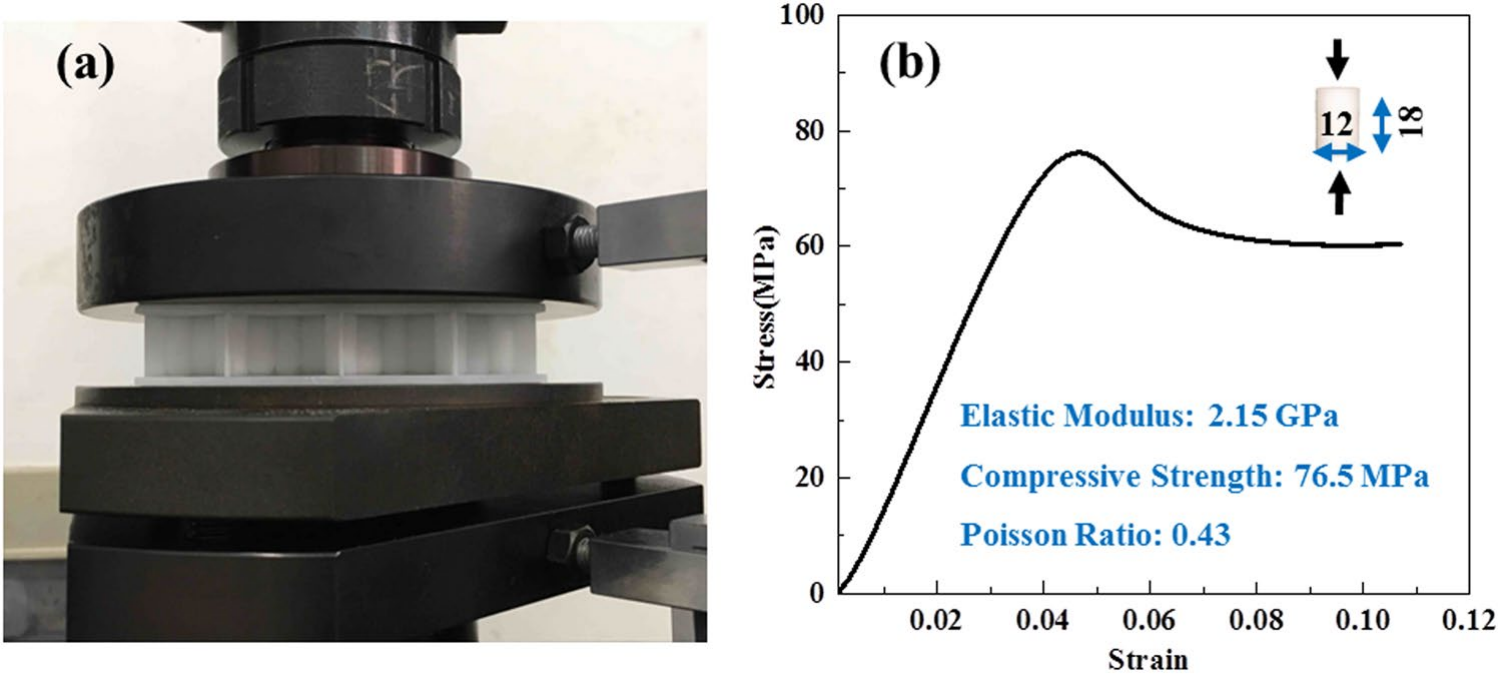
Experiment and material property. (**a**) The experimental process. (**b**) The constitutive curve of resin.

The dimensions of the plates were designed in accordance with the requirements for standard compressive test specimens^27^. Regarding the material, previously, experimental samples were produced using chopped-basalt-fiber-reinforced polymer (BFRP)^23^. However, when BEPs with hollow trabeculae are fabricated using this material, problems related to inhomogeneous fiber distributions and difficulties in template production arise during manufacture. Moreover, the sandwich plates that are currently used in various fields can be made of aluminum alloys, composite materials, plastics, paper or various other materials. The differences in the mechanical properties and failure modes of HPs and BEPs can be effectively compared because resin (DSM Somos 14120) is uniform, stable, and suitable for use in 3D printing with small errors. Therefore, resin was chosen for this experiment, and the test samples were created via 3D printing, and a ProJet model machine, manufactured by 3D Systems (South Carolina, U.S.), was used to print these experimental samples. The compressive test device was a T5105 electronic testing machine manufactured by MTS, shown in Fig. 4(a). The test models were subjected to displacement loading at a rate of 1 mm/min, with a sample size of 5. The material properties of the resin are illustrated in Fig. 4(b). The energy dissipation of unit volume of the core structure (Equation 1, hereafter referred to as the energy dissipation, and volume of the core structure *V*_*c*_ was used because the material density of BEPs and HPs are same) is used as the index to evaluate its energy dissipation capacity of two types of plates in this paper. The deformations of the upper and lower skins were neglected in this calculation because the core structure sustains the overwhelming majority of the deformation under compression.

1 $${U}_{c}=\frac{{\int }_{0}^{D}Fd\delta }{{V}_{c}}$$

where:

*U*_*c*_— the energy dissipation of unit volume of the core structure;

*F*— vertical load on the core structure;

*F* = *σA*, *σ*: stress in the core structure, *A*: cross sectional area of the core structure;

*δ*— vertical deformation of the core structure;

*δ* = *εh*, *ε*: vertical strain in the core structure, *h*: height of the core structure;

*D*— maximum deformation of the core structure;

*D* = *ε*_*max*_ *h*, *ε*_*max*_: maximum strain in the core structure;

V _*c*_— volume of the core structure.

## Results and Discussion

### Compressive properties of BEPs produced using the full integrated method

**Figure 5:**
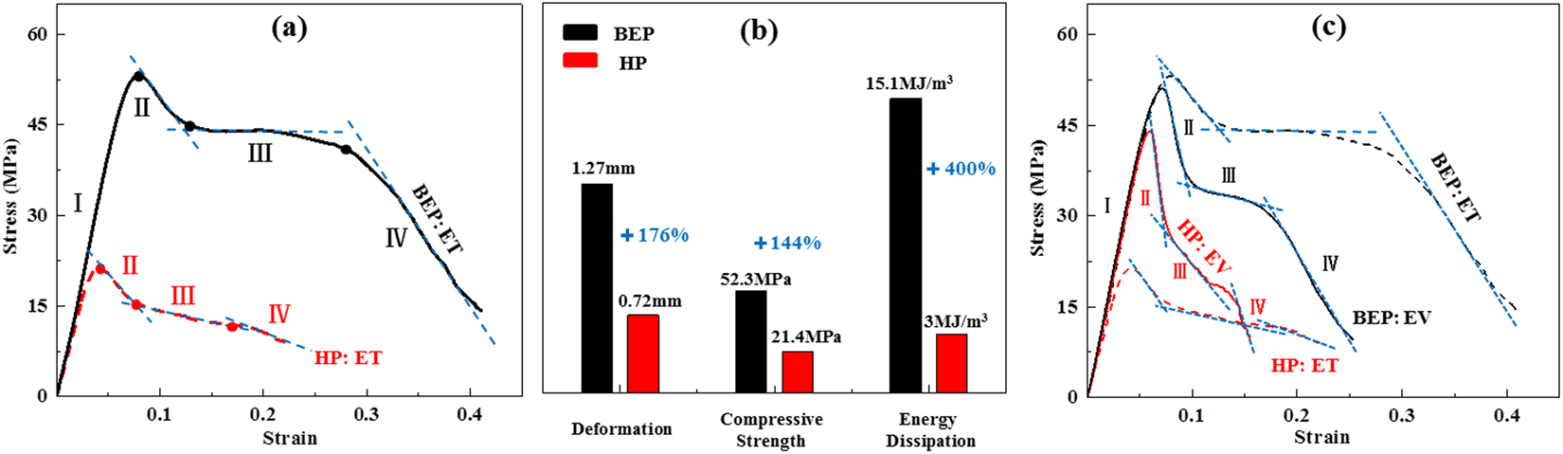
The experiment results of the two types of fully integrated sandwich plates. (**a**) The stress-strain curves of the core structures in the ET case. (**b**) The compressive properties of the two types of plates in the ET case. (**c**) The stress-strain curves of the core structures in the EV and ET cases. Where, the index of deformation in (**b**) is the value when those curves in (**a**) reach the position of compressive strength.

Figure 5(a,b) shows the results for the two types of fully integrated sandwich plate of equal thickness (ET). To facilitate comparison of the results, Fig. 5(c) shows the stress-strain curves for both EV and ET samples. It is evident from Fig. 5(a) that stage I is the elastic deformation for both sandwich plates. The compressive strength of the BEP is 2.44 times than that of the HP, and the corresponding deformation is 2.76 times higher (Fig. 5a,b). Stages II~IV of the curves are the plastic deformation stages. During these stages, for both the EV and ET samples, but especially in the latter case, the BEP curve during stage III is higher and extends significantly to the right, which shows a good plastic deformation capacity (Fig. 5c, BEP), whereas the HP curve is nearly at the lower left corner and decreases rapidly (Fig. 5c, HP). In other words, the stress-strain curves in Fig. 5(c) indicate that the BEP has not only a high compressive strength but also an excellent energy dissipation capacity, which of the BEP is 5.0 times that of the HP in the ET case, according to formula (1) (Fig. 5b).

**Figure 6:**
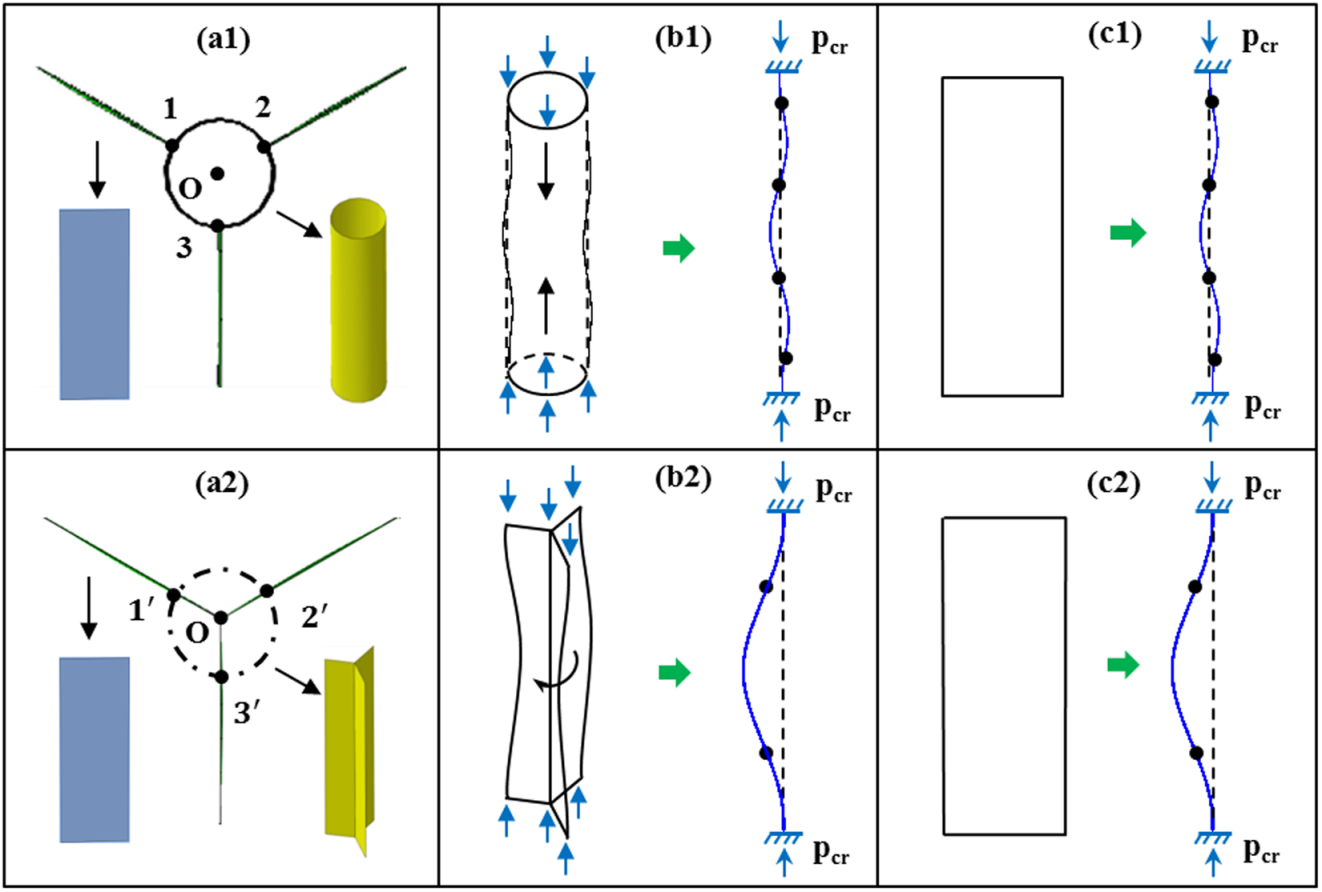
The deformation of the core structure of the sandwich plates. Upper row: BEP; lower row: HP. (**a**) The subdivision of the basic unit. (**b**) The column structure at the intersection of the honeycomb walls. (**c**) The honeycomb wall. The black point are the inflection point.

The first reason for these differences is that the triangular column at each intersection of honeycomb walls in the HP is a structure with an open section (as described in our previous paper^25^, a HP can be seen as two types of components, namely, honeycomb walls and triangular column structures, as shown in Fig. 6a2). The behavior of these open columns is dominated by torsional deformation, and a deformation curve characterized by a single half-wave is generated (Fig. 6b2,c2). By contrast, the trabeculae in the BEP have a closed section (Fig. 6a1) and a three half-wave convex deformation is generated (Fig. 6b1,c1), what’s more, the degree of which is far less than that of the HP. According to the theory of elastic stability^28^, the critical load on a thin plate that exhibits three half-wave deformation behavior is much higher than that on a plate that exhibits single half-wave. Meanwhile, the bearing capacity of a member that is undergoing compressive deformation is higher than that of one that is undergoing torsional deformation. Therefore, these results illustrate that the BEP has a higher strength and a higher elastic-plastic deformation capacity compared with the HP.

The second reason for the observed behavior lies in the difference between the EV and ET cases for the two types of plates. As mentioned previously, in the EV case, the wall thicknesses of the BEP and HP are 1 mm and 1.4 mm, respectively, whereas the wall thicknesses of both plates investigated in the this study are 1 mm (ET). According to the theory of elastic stability^28^, when the elastic modulus of such a plate is E, the Poisson’s ratio is ν, the buckling coefficient is k, and the wall thickness is t, the yield stress *σ*_*cr*_ of the thin plate can be calculated using formula (2):

2 $${\sigma }_{cr}=\frac{{p}_{cr}}{t}=\frac{k}{12(1-{\nu }^{2})}\times \frac{{\pi }^{2}E}{{(L/t)}^{2}} \sim \frac{1}{{(L/t)}^{2}}$$

where L is the loaded edge length, which is equal to the length of the honeycomb walls in the core structure (mm).

According to formula (2), the thin plate buckling stress is inversely proportional to the square of the ratio between the loaded edge length and the thickness of the plate (L/t). The value of L for the HP in the EV case^25^ is only 8% higher than that for the HP in the ET case, whereas t is increased by 42%. Thus, compared with the HP: ET (Fig. 5c, the dotted line on the bottom labeled HP: ET), the compressive strength of HP: EV is considerably improved (Fig. 5c, the solid line labeled HP: EV). However, the plastic deformation and energy dissipation are not significantly improved, especially in stage II and III, where the curve exhibits its fastest and largest decline. This means that although the compressive strength of the HP: EV can be improved by increasing the wall thickness by 42%, the energy dissipation are not enhanced because this structure is still inferior to that of the BEP. By contrast, in the ET case, the core volume of the BEP is increased by 42% compared with the HP, whereas the total volume is increased by only 9%. As shown in Fig. 5(c), the compressive strength is 2.44 times that of the HP, and the energy dissipation capacity is increased by a factor of 5.0.

In fact, the skins of the sandwich plates that are used in practical engineering are much thicker than the walls of the core structure. Therefore, the added material cost for the BEP in the ET case is very small. However, the compressive strength and energy dissipation capacity are improved significantly, which is of great benefit for engineering applications. This study shows that over tens of millions of years of evolution, the beetle has developed an organic buffering structure that possesses a much better mechanical properties than those of honeycomb, which is commonly used as a lightweight and high-strength buffering structure; consequently, this makes it possible for the BEP to be a new generation of functional-structural materials.

### Influence of single integrated method on compressive properties of BEP

**Figure 7:**
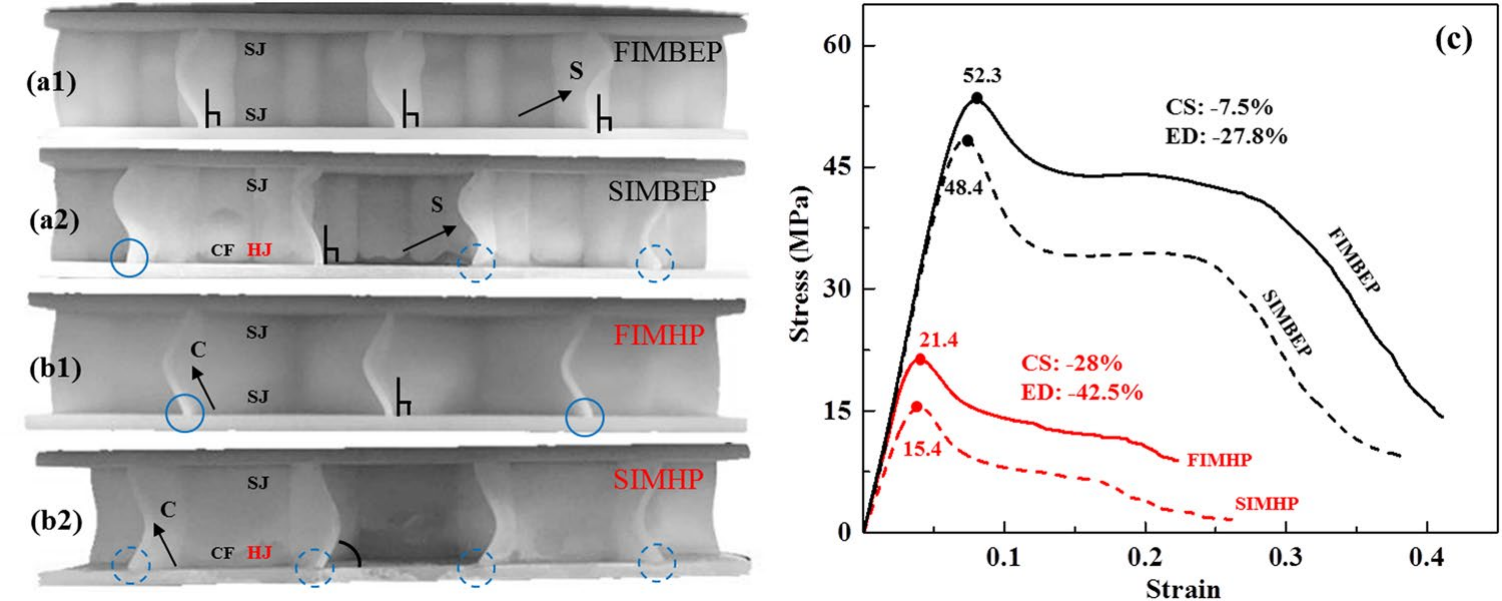
The experiment results on sandwich plates prepared using two different molding methods. (**a**,**b**) The deformation of the BEPs and HPs, respectively, at the end of stage II. (**c**) The strain-stress curves of the core structures. In this paper, FIMBEP and FIMHP denote sandwich plates produced using the full integrated method, whereas SIMBEP and SIMHP denote sandwich plates produced using the single integrated method. CF denotes a cement face; SJ denotes the solid joint; HJ denotes the hinge joint; CS denotes the compressive strength; ED denotes the energy dissipation. Dotted circles indicate instances of degumming or stripping between the honeycomb walls and the lower skin; vertical lines indicate right angles between the honeycomb walls and the lower skin; and solid circles indicate instances of intermediate deformation between the degumming case and the right-angle case, in which the core structure does not separate from the skins but not the honeycomb walls meet the skins at right angles.

Figure 7 shows the results of compression experiments on the two types of sandwich plates produced using the two different molding methods, FIM and SIM, respectively. It can be seen from Fig. 7(a,b) that when different molding methods are used, the angles between the honeycomb walls (the parts closest to the skins, approximately 1–2 mm) and the lower skin are different: the phenomenon of degumming or stripping between the core structure and the lower skin appears only on the cement faces (CF), especially in the regions indicated by the four dotted circles on the honeycomb plates (Fig. 7b2), an obvious inclination of the honeycomb walls is produced at an acute angle; whereas the walls meet the lower skins mostly at right angles when the plates produced using the FIM (Fig. 7a1,b1, vertical lines), with one exception of the cement faces in the BEP produced via the SIM. According to a mechanical constraint analysis, these findings can be attributed to the different connection types: solid joint (SJ) are formed under the strong constraint conditions imposed by the FIM, whereas hinge joint (HJ) form because of the phenomenon of degumming or stripping that appears at the cement faces created in the case of the SIM, which are subject to a weak constraint condition (Fig. 7a2,b2).

The cement faces can easily suffer from degumming or stripping, as mentioned above, under the weak constraint conditions imposed by the SIM. Thus, for the SIMBEP, this phenomenon was denoted by dotted circles in Fig. 7(a2). However, because the lateral deformation degree of core structure of the BEPs is smaller than that of the honeycomb plates, there is a solid circle and a right angle appearing in Fig. 7(a2). What’s more, these right angles can be maintained in BEP when the FIM is used (strong constraints, vertical lines in Fig. 7a1). By contrast, in the HPs, the lateral deformation degree of the honeycomb walls and triangular columns is greater. Thus, even when the FIM is used, an intermediate form of deformation is observed in which not the walls of the core structure meet the skins at right angles, although the core structure does not separate from the skins (Fig. 7b1, solid circles). Regarding the images of the BEP and HP produced using the SIM, the former is marked with only two dotted circles, whereas the latter has four. This is because of the closed section of the trabeculae in the BEP, which consequently suffers a far lesser degree of lateral deformation compared with the distortion of the open triangular columns in the HP. Notably, the cross-sectional area of the trabeculae accounts for 78% of the cross area of the core structure, which can constrain and reduce the lateral deformation of the honeycomb walls to a large extent, as demonstrated by the experimental results, which show that the deformations of the honeycomb walls in the BEPs are mostly in the form of S curves, whereas the deformations in the HPs are in the form of C curves (Fig. 7a,b).

As seen from the stress-strain curves in Fig. 7(c), the compressive strengths of the BEP and HP are reduced by 3.9 MPa and 6.0 MPa, respectively, by the use of the SIM instead of the FIM. Although the compressive strength of the HP is smaller, the decline in the performance of the HP with SIM is more than 20%, greater than that of the BEP. In other words, for the BEP, the compressive strength and the energy dissipation decrease by 7.5% and 27.8%, respectively, whereas those of the HP decrease by 28% and 42.5%, respectively (Fig. 7c).

Thus, it can be seen that a BEP is an excellent example of a functional-structural material, and even when the SIM is used, the compressive strength of a BEP is still 3.1 times that of a HP with the same wall thickness, and the energy dissipation capacity is 6.3 times higher, because of the structural superiority of beetle elytra compared with honeycomb. Therefore, from the perspective of engineering applications, the SIM could be adopted to prepare BEPs before FIMBEPs are developed.

## Conclusions

To investigate the characteristics of compression, buffering and energy dissipation in the BEP, compression experiments were conducted in this study to compare the BEP with the HP, for plates constructed with the same wall thickness of the core structure and using different molding methods. The results are as follows:

1. Regardless of which molding method is used and whether the core structures of the BEP and HP are of equal volume (EV) or equal thickness (ET), the stage III stress-strain curve of the BEP shows a good plastic deformation capacity, particularly in the ET case. By contrast, for the HP, the curve drops sharply in stages II–IV.
2. Compared with the core structure of a HP, a closed-section hollow trabecula is located at each intersection of the honeycomb walls in a BEP, and the critical load, which exhibits a deformation pattern characterized by three half-waves, is much larger than that of the HP, which exhibits one half-wave deformation. The buckling stress of such a plate is proportional to the square of its wall thickness (t). Thus, for core structures with equal wall thickness, although the core structure volume of the BEP is greater than that of the HP by 42% (whereas the total volume increases by only 9%), the compressive strength increases by a factor of 2.44, and the energy dissipation capacity considerably increases by a factor of 5.0 when these two types of sandwich plates are prepared using the FIM.
3. At the end of stage II, degumming or stripping appears at the cement faces when the single integrated method (SIM) is used, whereas this phenomenon does not occur in the sandwich plates prepared using the full integrated method (FIM). This difference can be explained by mechanical constraint analysis: the FIM and SIM can be seen as imposing constraint conditions between the core structure and skins that result in solid joints and hinge joints, respectively. Nevertheless, even when the SIM is applied to prepare a BEP, its compressive strength is 3.1 times that of a HP manufactured in the same way in the ET case, and its energy dissipation capacity is greater by a factor of 6.3.

HPs, as typical lightweight, high-strength structures, have been widely applied in many fields ranging from transportation to aerospace engineering. However, with nearly identical material costs and in different manufacturing methods, BEPs offer a compressive strength and a dissipating performance that are several times those of HPs. Therefore, it is clearly demonstrated that this biological structure, which has evolved in the beetle since the age of the dinosaurs, is a buffering functional-structural material with a high compressive strength and energy dissipation capacity.
